# Assessment of the bifidogenic and antibacterial activities of xylooligosaccharide

**DOI:** 10.3389/fnut.2022.858949

**Published:** 2022-08-25

**Authors:** Zhongke Sun, Zonghao Yue, Erting Liu, Xianfeng Li, Chengwei Li

**Affiliations:** ^1^College of Biological Engineering, Henan University of Technology, Zhengzhou, China; ^2^Institute of Food and Drug Inspection, Zhoukou Normal University, Zhoukou, China; ^3^Henan Heagreen Bio-technology Co., Ltd., Zhoukou, China

**Keywords:** xylooligosaccharide, dose effect, degree of polymerization, bifidogenic activity, antibacterial activity

## Abstract

Xylooligosaccharide (XOS) is an attractive prebiotic mainly due to its bifidogenic effect. However, commercial XOS with different compositions is often applied in the food industry at different doses without specifications. In this study, we evaluated the bifidogenic activity of XOS at different doses with either mixtures or pure fractions with different degrees of polymerization (DP), using three strains of *Bifidobacterium* spp., including *B. breve* ATCC 15700, *B. bifidum* ATCC 29521, and *B. animalis* subsp. *lactis* HN019. Three growth indicators showed strain-specific bifidogenic activity of XOS, and the activity was both dose- and fraction-dependent as only certain fractions stimulated significant growth. Adding 0.25% XOS (w/v) also promoted increase in total bifidobacterial population of rat fecal samples fermented *in vitro*. Albeit the antibacterial activity of XOS fractions can be demonstrated, significant growth inhibition can only be achieved when 4.0% XOS mixture was added in *Staphylococcus aureus* ATCC 6538 pure culture. In contrast, in the presence of *B. lactis* HN019, 1.0% XOS showed significant antibacterial activity against *S. aureus* ATCC 6538 in milk. In addition, RNA sequencing suggested downregulation of genes involved in *S. aureus* ATCC 6538 infection, pathogenesis, and quorum sensing, by XOS. In conclusion, the report urges scientific specifications on XOS chemistry for its effective application as a novel food ingredient or functional food and provides novel insights into its bifidogenic and antibacterial activities.

## Introduction

The International Scientific Association for Probiotics and Prebiotics (ISAPP) has categorized xylooligosaccharide (XOS) as a prebiotic or prebiotic candidate (1). Potential health-related effects of XOS include improved stool frequency and consistency, reduced carcinogen formation in the colon, balanced lipid and glucose metabolism, enhanced immune function, and anti-oxidation or antimicrobial activities (2, 3). However, as a prebiotic, the principal effect of XOS on human is stimulating the growth of bifidobacteria, termed the bifidogenic effect (4, 5). Increase in bifidobacteria population is widely recognized as a beneficial effect due to their correlation with many positive health outcomes (6). A study *in vitro* demonstrated that XOS produced the highest increase in cell numbers of bifidobacteria compared to other prebiotics over 24 h of batch culture through fluorescent *in situ* hybridization (7). In pure cultures, XOS was fermented with high specificity by *Bifidobacterium animalis* subsp. *lactis* (*B. lactis*) strains (8). Therefore, XOS was considered a cost-effective prebiotic and has attracted great interest, especially in the dairy industry.

Although XOS is a widely commercialized prebiotic, it is a mixture of xylose oligomers made up of units with variable degrees of polymerization (DP, average DP ≤ 20 in commercial XOS) and has been produced by different methods (9). Different chemical/structural characteristics of XOS are indefinitely affecting the bioactive properties/effects of XOS as a functional food (10). However, the exact bifidogenic effect of different fractions in commercial XOS is still elusive. For example, non-substituted XOS and arabino-XOS can be fermented more quickly than acetylated XOS and XOS containing a 4-*O*-methylglucuronic acid group by fecal inocula (11). Compared to XOS fractions with larger DPs, fractions with an average DP 4–14 increased bifidobacteria population more significantly, suggesting bifidobacteria preferred XOS components with relatively lower molecular weight (12). The short-chain XOS (DP 2–5) improved bifidobacteria replication over medium-chain or long-chain XOS did, in the first stage of fermentation (13). More precisely, XOS fractions with average DP 3–4 promoted faster growth of *B. adolescentis* than that with average DP 5–6 did (14). Differently, in another study, substituted XOS mixtures with both smaller (DP 4–6) and larger DPs (DP 9–21) stimulated bifidobacteria count to a similar level of a commercial XOS with DP 2–6 did (15). Considering that the composition is vital for commercial XOS quality control and desired nutrition, comparison of their bifidogenic activity becomes important and necessary.

As a food ingredient, dose is another important parameter for the anticipated nutrition of XOS during application. The bifidogenic effect of XOS was demonstrated in several studies with different fermentation models at different doses at present. As early as 30 years ago, Okazaki et al. had already reported that 5 g/day XOS selectively promoted the growth of bifidobacteria and helped to maintain the fecal water content within normal range in human (16). Later, it was shown intake of XOS at doses of only 1.4 g/day for 8 weeks could significantly increase bifidobacteria counts in healthy adults (17). In a simulated colon model, 0.5 g/day XOS resulted in significant increases of bifidobacteria (18). However, intake of 0.5 g/day XOS only increased the relative abundance of *Bifidobacterium* spp. in the fecal samples by qPCR analysis, and the results cannot be confirmed by sequencing data in rat (19). On agar plate, the growth of 35 *Bifidobacterium* strains was stimulated by XOS at a dose of 6.25 mg/ml (20). In the human fecal samples, adding 1.25% XOS resulted in a significant increase in *Bifidobacterium* spp. operational taxonomic units (OTU) from 0.67 to 5.22, after *in vitro* fermentation for 24 h (21). As seen, XOS was added at different doses, and inconsistent bifidogenic effect was shown. Therefore, the determination of the relationship between XOS doses and its bifidogenic activity is another prerequisite for its effective application.

The antibacterial activity of XOS is also important for shaping gut microbiota and enhancing food safety (22). By testing the inhibition zone on agar plates, an *in vitro* study demonstrated high antimicrobial activity of XOS against *Klebsiella pneumoniae, Enterococcus faecalis, Bacillus thuringiensis*, and *Pseudomonas aeruginosa* (23). *In vivo*, the antimicrobial activity of XOS can be even enhanced, for example, decreasing the viable Enterococcus, Enterobacter, and Clostridia after metabolism by probiotics (24). However, the effect is often variable among different XOS products, due to the structural complexity and chemical heterogeneity (25). In addition, little information is available on its antibacterial mechanisms, especially which fractions are most effective and how XOS counteracts those gram-positive pathogens that widely presented on food chains.

To address the aforementioned questions, this manuscript evaluated the relationship between XOS doses, fractions, and these activities *in vitro*. Using three strains/species of *Bifidobacterium*, the bifidogenic activity was demonstrated by comparison of their fermentability to either xylose or XOS mixture under different doses of XOS (from 0 to 2.0%) or fractions of different DPs (DP 2, 3, 4, 5, and 6) in pure cultures at first. The bifidogenic effect was also quantified using rat fecal samples with 0.25% XOS. Then, the antibacterial activity of XOS was probed by growth inhibition of *Staphylococcus aureus* ATCC 6538 (*S. aureus* ATCC 6538), a reference strain for the disinfectant susceptibility test (26). The antibacterial activity was also tested in milk by adding both *B. lactis* HN019 and XOS. At last, the impact of XOS on *S. aureus* ATCC 6538 gene expression was investigated by RNA sequencing.

## Materials and methods

### Chemicals, bacterial strains, and growth conditions

D-xylose was purchased at 98% purity (Cat. No. D856756, Macklin Inc., Shanghai, China). The XOS and its pure fractions (XOS_2_, XOS_3_, XOS_4_, XOS_5_, and XOS_6_) were produced by a commercial supplier (Henan Heagreen Bio-technology Co., Ltd., Zhoukou, China). The detailed chemical composition and purity data can be found in Supplementary Table 1. All XOS and its fractions were dissolved separately in distilled water at a final concentration of 50% (w/v). The solutions were filter-sterilized (0.22 μm, Millipore, United States) and stored at 4°C within 1 week of preparation. Bacterial strains, *Bifidobacterium breve* ATCC 15700 (*B. breve* ATCC 15700) and *Bifidobacterium bifidum* ATCC 29521 (*B. bifidum* ATCC 29521), were purchased from Guangdong Institute of Microbiology Culture Center (GIMCC, Guangzhou, China). *Bifidobacterium lactis* HN019 was provided by Dupont Co. Ltd. (Shanghai, China). All bifidobacterial strains were propagated under anaerobic conditions at 37°C as described elsewhere (27). Strain *S. aureus* ATCC 6538 was purchased from a commercial supplier (Baofeng Com. Ltd., Shanghai) and was used as an indicator strain for antibacterial activity test. The strain was grown in standard Luria–Bertani (LB) broth by shaking at 150 rpm under 37°C.

### Test of xylooligosaccharide fermentation, relationship between dose, fractions, and bifidogenic activity

A basic biochemical broth (BBB) was manually prepared with minor modification of the Biochemical Basis Medium of *Bifidobacterium* (Cat. No. HB8521, Qingdao Hopebiol Co., Ltd., China). The medium supports poor growth of bifidobacteria but does not contain any sugar. The BBB medium contained 10 g bacterial peptone, 5 g tryptone, 1 g Tween 80, 0.2 g L-cysteine, 0.2 g MgSO_4_, 20 mg bromocresol purple, 10 mg NaCl, 6.7 mg MnSO_4_, and 1 mg FeSO_4_ per liter. The broth was used as a basis for the following tests. For fermentation ability test, 0.25% (final concentration, w/v) xylose or XOS was added to the broth. For XOS dose test, 0, 0.125, 0.25, 0.5, 1.0, and 2.0% XOS were added into 5 mL broth. For XOS fraction test, xylose and XOS fractions of DP 2, 3, 4, 5, and 6 were added separately at 0.25%. Each strain cultured overnight was inoculated into different tubes to an initial OD600 = 0.05 and then incubated anaerobically at 37°C for 24 h. To quantify the biomass of bacteria, three indicators including optical density, count number, and *groEL* gene copy number were detected. Briefly, the fermentation broth was fully vortexed, and optical density at 600 nm (OD600) was monitored using a microtiter reader (SpectraMax i3x, Molecular Devices, United States). Growth in the presence of sugars was shown as the relative OD600 (Rel. OD600) to that in the absence of sugar. Diluted culture broth was plated in triplicate on MRS agar (Cat. No. HB0384, Qingdao Hopebiol Co., Ltd., China). The count number was calculated by cfu/mL in original culture fluids. The copy number of *groEL* was quantified by qPCR using a pair of bifidobacterial genus-specific primers (28). Compositions and parameters for qPCR reaction were same as our previous report using SYBR green as the fluorescent dye (29). The relative copy numbers of *groEL* were analyzed by the 2^–ΔΔ Ct^ method, normalized to that in BBB broth without any sugar.

### Collection of rat fecal samples and *in vitro* fermentation

The fresh fecal samples were collected by a company from six healthy female rats (adult female Sprague-Dawley rats, body weights ranging from 250 to 280 g) fed under specific pathogen-free conditions according to standard guidelines (Servicebio Technology Co., Ltd., Wuhan, China). The feces were added into sterile 15 mL tubes, placed on an anaerobic chamber, and transported at 4°C to the laboratory. The fecal samples were inoculated into the gut microbiota medium (GMM) as described with minor modification (30). Briefly, the fecal samples were suspended in pre-reduced PBS with 0.1% cysteine (10 mL/g feces) by vortexing for 5 min. The suspension of 0.5 mL was used as microbial inocula after standing at room temperature for 5 min to permit large insoluble particles to settle to the bottom of the tube. After anaerobic incubation for 72 h at 37°C in 10 mL GMM in the presence or absence of 0.25% XOS, all microbial cells were collected by centrifugation at 10,000 g for 2 min and then washed twice with double-distilled water (ddH_2_O). The dilute cell suspension was used for plating MRS agar. Genomic DNA was extracted from cell pellets using a Universal Genomic DNA Kit (Cat. No. CW2298S) purchased from CoWin Biosciences (Beijing, China). The genomic DNA was used as template to quantify the relative copy number of *groEL* by qPCR as described above.

### Antibacterial activity assay of xylooligosaccharide and its fractions on *Staphylococcus aureus* ATCC 6538

The antibacterial activity was first tested against strains *S. aureus* ATCC 6538 by evaluating growth inhibition in pure culture. Briefly, the strain was grown in 5 mL LB broth supplemented with different doses of XOS (0, 0.25, 0.5, 1.0, 2.0, and 4.0%, w/v) under 37°C for 24 h. The cell suspension was collected at different time points. To test the effect of different fractions (xylose, XOS_2_, XOS_3_, XOS_4_, XOS_5_, and XOS_6_), the strain was grown in 200 μL LB broth supplemented with 1.0% pure fractions using sterile microplates for 24 h. Growth inhibition was determined by reading OD600 as described above. To further study the antibacterial activity of XOS in complex food matrices, it was added into milk inoculated with *S. aureus* ATCC 6538. Precisely, ultra-heat treated pure milk was filled into four sterilized glass tubes (10 mL in each) added with/without 1.0% (w/v) XOS and in the presence/absence of 1.0% (v/v) *B. lactis* HN019 cell suspension (10^6^ cfu/mL in ddH_2_O). All tubes were incubated at 25°C for 7 days. The samples (1 mL) were collected at day 2 and day 7 after fully vortexing. The diluted samples were plated on LB agar in triplicate. The relative count number was used as another indicator for evaluating antibacterial activity.

### Analysis of xylooligosaccharide impacts on *Staphylococcus aureus* ATCC 6538 by RNA sequencing

Overnight culture fluids of *S. aureus* ATCC 6538 were inoculated (1% v/v) into LB broth in the absence (group N) or presence of 1.0% XOS (group X). After 12 h shaking, the cells were harvested from triplicate cultures by centrifugation at 10,000 g for 2 min at 4°C. TRIzol^®^ reagent (Thermo Fisher) was used for total RNA extraction. The concentration was measured by Nanodrop2000, and the purity was detected by agarose gel electrophoresis. The cDNA library was constructed using the TruSeq™ Total RNA Library Prep Kit (Illumina, San Diego, CA, United States). The RNA sequencing was carried out in a NovaSeq 6000 system (Illumina, United States) by a commercial company (Majorbio Biotech Co., Ltd., Shanghai, China). All clean reads were aligned with the reference genome (accession: NZ_CP020021.1) annotated by BLASTX alignment in different databases. Differentially expressed genes (DEGs) between the control (N) and the treatment (X) groups were screened with the default threshold of a false discovery rate *p* < 0.05 and | log2FC| ≥ 1. Enrichment analyses of Gene Ontology (GO) and Kyoto Encyclopedia of Genes and Genomes (KEGG) pathways for DEGs were also conducted on a commercial server.^1^ The raw data had been deposited in the NCBI Sequence Read Archive (SRA) with the accession numbers SRR17670774-SRR17670779 under BioProject PRJNA798775.

### Statistical analysis

Statistical analysis was carried out using SPSS 19.0 (SPSS Inc., United States). Data were expressed as mean ± standard deviation (SD). Significant differences were analyzed by *t*-test and Tukey’s one-way analysis of variance (ANOVA) when necessary. The values of *p* < 0.05 were considered as statistically significant.

## Results and discussion

### The bifidogenic activity of xylooligosaccharide varies among different strains

Relative to the growth in BBB, all tested bifidobacterial strains grown in the presence of XOS reached to a higher biomass, indicating effective fermentation of XOS (Supplementary Figure 1). However, only *B. lactis* HN019 used xylose as effectively as XOS. The other strains, *B. breve* ATCC15700 and *B. bifidum* ATCC 29521, grow to significantly lower level when using xylose as sole carbohydrate. Nevertheless, obvious color change (from blue to yellow after incubation) of the fermentation broth suggested fermentation of xylose by *B. bifidum* and *B. breve* as well. All tested strains grew much better in XOS than in BBB medium suggesting XOS stimulated growth, which is consistent with the previous studies (8, 18).

Critically, the bifidogenic effect of an oligosaccharide should be compared to its corresponding monosaccharide, other than in a medium without sugar. Therefore, the bifidogenic effects of 0.25% XOS on different strains were further calculated to those in 0.25% xylose. Relative to the growth in xylose, *B. lactis* grew to similar levels when using XOS as substrate, as all relative values were close to 1. In contrast, the relative values of all three indicators are much higher in *B. breve* and *B. bifidum* (Supplementary Figure 2). The results indicated the bifidogenic effect of XOS varies among different strains/species, which might be due to different preferences to XOS and having different metabolism pathways (5, 31). The utilization of XOS includes two processes, internalization and transformation. Generally, bifidobacteria can internalize carbohydrates by ATP-dependent ABC transporters and PEP-PTS systems, with the former as the primary transport systems (32). However, according to a transcriptomic study, several transporters can be induced by XOS in *B. adolescentis* (33). Therefore, different species may use different transporters that have different importation efficiencies or binding affinities. After internalization, carbohydrates can then be hydrolyzed, phosphorylated, deacetylated, and/or transglycosylated by dedicated intracellular enzymes. Glycosyl hydrolases appear to be the major group of enzymes for bifidobacteria (34). Carbohydrates were ultimately transformed to phosphoenolpyruvate through glycolysis and pentose conversions during the fermentation by bifidobacteria and further involved in the tricarboxylic acid cycle (35). For example, based on a proposed model for the catabolism of XOS in *B. lactis* BB-12, the strain utilizes an ABC (ATP-binding cassette) transport system (probably for oligosaccharides) to bind XOS on the cell surface and transport them into the cell. XOS is then degraded intracellularly through the action of xylanases and xylosidases to d-xylose, which is subsequently metabolized by the bifido shunt, also termed the F6PK pathway (36).

In fact, strain-specific growth promotion of *Bifidobacterium* spp. by XOS had already been recognized in the previous studies (8, 37). In addition, the relative values of three indicators for each strain are different possibly due to different sensitivities of these methods, for example, the value for the relative count number is much higher than the values for both OD600 and *groEL* copy number.

### The bifidogenic activity of xylooligosaccharide is both dose- and fraction-dependent

**FIGURE 1:**
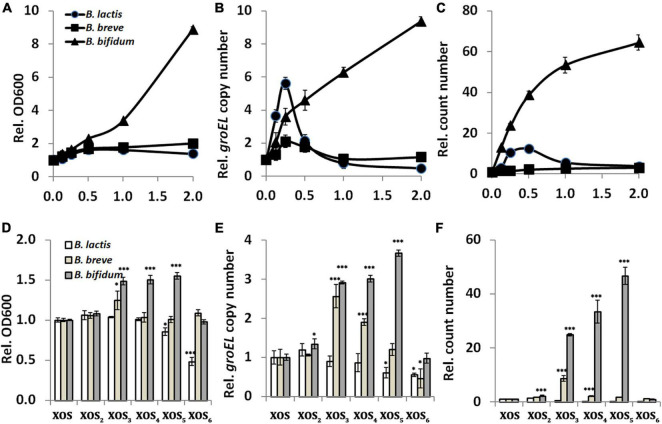
Relationship between xylooligosaccharide (XOS) doses or fractions and its bifidogenic activity. **(A,D)** Growth assayed by reading OD600, **(B,E)** *groEL* gene copy number assayed by qPCR, **(C,F)** cell count number by counting colonies on MRS agar. In panels **(A–C)**, strains of *Bifidobacterium* spp. were inoculated into the basic biochemical broth supplemented with XOS at different doses (0, 0.125, 0.25, 0.5, 1.0, and 2.0%, w/v, final concentration). Data were normalized to that of the samples collected from medium supplemented with xylose. In panels **(D–F)**, strains of *Bifidobacterium* spp. were inoculated into the basic biochemical broth supplemented with 0.25% XOS or its fractions (XOS_2_, XOS_3_, XOS_4_, XOS_5_, and XOS_6_). Data were normalized to that of the samples collected from medium supplemented with XOS. All samples were collected after 24 h incubation. All data were mean of three independent experiments assayed in triplicates. *B. lactis, Bifidobacterium animalis* subsp. *lactis* HN019; *B. breve, Bifidobacterium breve* ATCC 15700; *B. bifidum, Bifidobacterium bifidum* ATCC 29521; *p < 0.05; ^***^p < 0.001.

To determine how much XOS should be added during application, the dose–response experiment was performed. For parallel comparison, the BBB medium was used as control, and the values of growth indicators in it were used for normalization. Our study with three strains *in vitro* showed significant effects of dose (Figures 1A–C). Constant increase in the values suggested a positive relationship between the bifidogenic activity and XOS dose (0–2%) in *B. bifidum* ATCC 29521. For *B. lactis* HN019 and *B. breve* ATCC 15700, dose has limited influence on the bifidogenic effect, and growth tends to reach similar levels in both xylose and XOS, albeit inconsistent data among three indicators. This can be a reason why a previous study argued that the daily dose is not a determinant of the bifidogenic effect *in vivo* (4). As one of the most widely applied strains in dairy product, *B. lactis* HN019 grows well in the medium when absent or present of either xylose or XOS (Supplementary Figure 1). However, agreed to the previous report, *B. breve* ATCC 15700 grows poorly in medium supplemented with both xylose and XOS, even it is widely presented in the digestive and urinary tract of human (38). Although it was speculated that the magnitude of the bifidogenic effect is mainly influenced by the number of bifidobacteria present in the colon before supplementation with prebiotics, dose affected the bifidogenic activity in *B. bifidum* ATCC 29521 in our *in vitro* study using pure culture, because stronger growth promotion was demonstrated when more XOS was added into medium (31). As a common isolate from human feces, *B. bifidum* ATCC 29521 is used as a probiotic to maintain healthy gut microbiota and to allow for normal digestion. In fact, a long-lasting bifidogenic effect on *B. bifidum* ATCC 29521 cultures has also been demonstrated by using other oligosaccharides (39, 40). In line with the previous observation, *B. bifidum* preferred XOS as substrate compared to xylose suggesting a specific transport system for the oligosaccharide over the monomer (41).

To determine mainly which fraction responded to the bifidogenic activity of XOS, growth was monitored in the presence of different pure fractions. For *B. bifidum* ATCC 29521, fractions including XOS_3_, XOS_4_, and XOS_5_ resulted in much higher growth than XOS mixture, indicating they are the major stimulators (Figures 1D–F). For *B. breve* ATCC 15700, mainly XOS_3_ has stronger bifidogenic effect than XOS has. The results are partially agreed to the previous studies, which showed preference of XOS_3_ and XOS_4_ and a remarkable ability to utilize XOS_2_ and XOS_3_ as the major fractions by *B. adolescentis* (13, 14, 42). However, it seems only XOS_2_ slightly enhanced the growth of *B. lactis* HN019 in this study, which agreed to a report in pure cultures (8). XOS fractions with larger DPs (mainly XOS_5_ and XOS_6_) do not stimulate but significantly inhibit growth of *B. lactis* HN019 in this study. This is inconsistent with a previous study performed in a colon simulator model, in which increased levels of *B. lactis* were measurable with XOS compounds that had larger DPs (18). Fermentation models and evaluating methods might raise these differences, as shown in this report and other previous studies (16–19).

### Xylooligosaccharide increases fecal total bifidobacteria population

**FIGURE 2:**
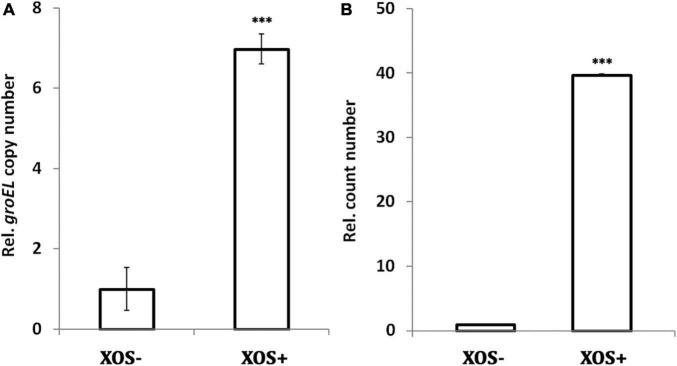
Bifidogenic effect of xylooligosaccharide (XOS) on rat fecal microbiota. **(A)** *groEL* gene copy number assayed by qPCR, **(B)** cell count number by counting colonies on MRS agar. The fecal samples were inoculated in GMM broth or GMM supplemented with 0.25% XOS. All samples were collected after 72 h incubation. All data were mean of six rats assayed in triplicates. Data were normalized to that of the samples collected from GMM medium in the absence of XOS. Data were analyzed by Tukey’s one-way analysis of variance (ANOVA). ^***^p < 0.001.

To confirm whether the bifidogenic activity of XOS in pure cultures is reproducible in complex cultures, rat fecal samples were collected for *in vitro* fermentation. After incubation of fecal inocula for 72 h, the gene copy of *groEL* is 6-fold higher in GMM supplemented with 0.25% XOS than in GMM alone, suggesting the bifidogenic effect of XOS is reproducible in a complex environment (Figure 2A). The result is in line with a previous study that showed roughly 6.8-fold increase in the *Bifidobacterium* spp. operational taxonomic unit in human fecal samples after *in vitro* fermentation in the presence of 1.25% XOS (21). XOS also increased bifidobacteria populations of human fecal inocula after *in vitro* fermentation, as reflected by fluorescent *in situ* hybridization (15). Contrasting to these culture-independent molecular detection methods, the traditional culture-dependent method showed stronger growth promotion of *Bifidobacterium* spp. by XOS. As shown, nearly 40-fold more cells were obtained on MRS agar (Figure 2B). Taken together, XOS has bifidogenic activity on gut microbiota, and the stimulation effect in bifidobacteria may be stronger *in vivo* than *in vitro*.

### Xylooligosaccharide has antibacterial activity against *Staphylococcus aureus* ATCC 6538

**FIGURE 3:**
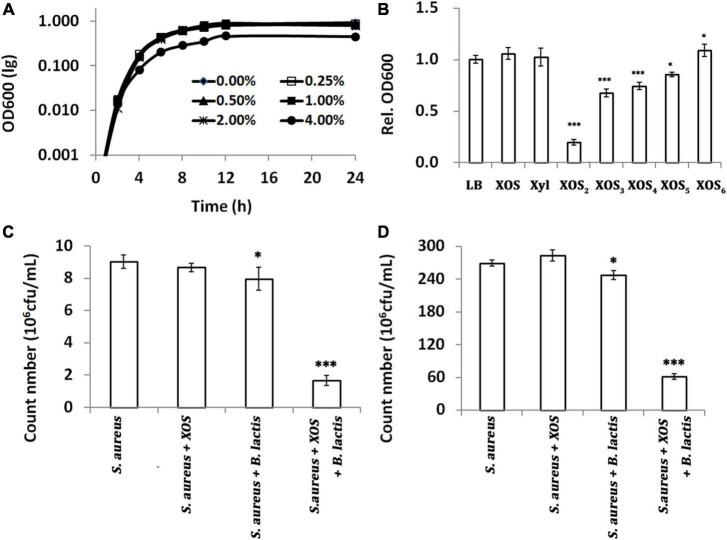
Antibacterial activity of xylooligosaccharide (XOS) and its fractions against *Staphylococcus aureus* ATCC 6538. **(A)** Growth curves under different doses of XOS, **(B)** growth inhibition by 1.0% different XOS fractions, **(C)** count number in milk after 2 days, **(D)** count number in milk after 7 days. In panels **(A,B)**, *S. aureus* was grown in LB broth supplemented with different doses or fractions of XOS. In panels **(C,D)**, *S. aureus* was grown in milk supplemented with 1.0% XOS, *B. lactis* HN019, or both. The samples in panels **(B–D)** were collected at 24, 48, and 168 h. All data were mean of three independent experiments assayed in triplicates. Data in panel **(B)** were normalized to that of the samples grown in LB, and data in panels **(B–D)** were analyzed by Tukey’s one-way analysis of variance (ANOVA) when necessary. *S. aureus, Staphylococcus aureus* ATCC 6538; *B. lactis, Bifidobacterium animalis* subsp. *lactis* HN019; *p < 0.05; ^***^p < 0.001.

Although the anti-pathogenic potential of XOS has been demonstrated by agar diffusion *in vitro* or microbial abundance analysis *in vivo*, there is no further study of its antibacterial activity thereafter, especially in food matrices (23, 43). Here, the antibacterial activity of XOS was measured by growth inhibition of *S. aureus* ATCC 6538, a gram-positive reference strain. In batch cultures reflected by OD600, dose has great impact on the antibacterial activity of XOS as only 4% XOS can significantly inhibit the growth, and no growth inhibition can be demonstrated when less than 4% XOS was added into medium (Figure 3A). To probe which fractions inhibited the growth, pure fractions were added, respectively, at a lower dose (0.25%, w/v) for 24 h fermentation. Contrasting to grown in LB broth, XOS fractions with smaller DPs (XOS_2_ and XOS_3_) showed stronger antibacterial activities (Figure 3B). As prebiotics are often added in combination with probiotics, the antibacterial activity of XOS was tested in milk at room temperature, in the presence of *B. lactis* HN019. In line with that in pure culture, 0.25% XOS did not influence the growth of *S. aureus* ATCC 6538 in milk. *B. lactis* HN019 alone slightly decreased the count number of *S. aureus* ATCC 6538. However, the addition of 0.25% XOS in the presence of *B. lactis* HN019 sharply decreased the count number of *S. aureus* ATCC 6538 (Figure 3C). The data also indicated efficient conversion of XOS by *B. lactis* HN019, to more deleterious substances to *S. aureus* ATCC 6538 (24). Deleterious substances might be short-chain fatty acids (SCFAs) that inhibit the growth of pathogenic organisms (44). It was found that these SCFAs not only inhibit growth by disrupting intracellular pH homeostasis, but also modulate gene expression of pathogens (45, 46). After 1 week, the count number is still significant lower (Figure 3D). Therefore, adding XOS may help improve shelf life, considering probiotics are widely added in milk.

### Xylooligosaccharide downregulates genes involved in *Staphylococcus aureus* ATCC 6538 pathogenesis

**FIGURE 4:**
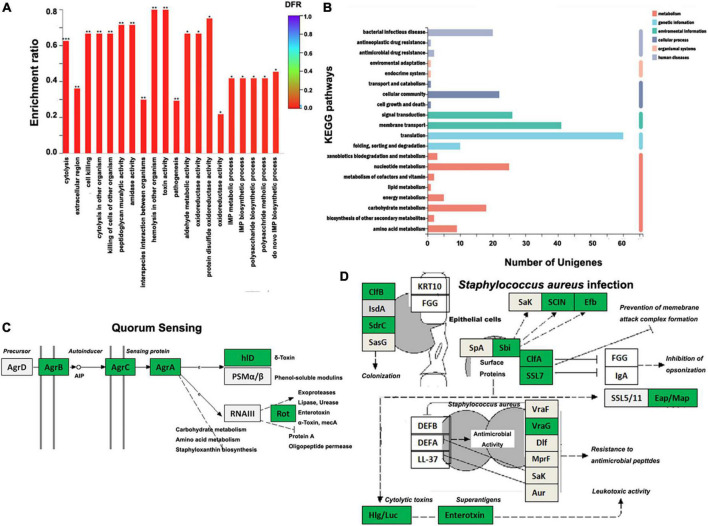
Influences of xylooligosaccharide (XOS) on *Staphylococcus aureus* ATCC 6538 gene expression. **(A)** GO enrichment analysis of the downregulated genes, **(B)** histogram of the top 20 KEGG pathways enriched by differently expressed genes (DEGs), **(C)** KEGG pathway of the quorum sensing (adapted from map02024), **(D)** KEGG pathway of *S. aureus* infection (adapted from map05150). *S. aureus* ATCC 6538 was inoculated into LB broth in the absence/presence of 1.0% XOS. In panels **(C,D)**, genes in green box are downregulated; genes in light gray box are expressed without significant difference. AIP, auto-inducing peptide; *p < 0.05; ^**^p < 0.01; ^***^p < 0.001.

RNA sequencing of six samples obtained 25.94 Gb clean data, with an average above of 3.82 Gb and raw Q30 > 94.62%. Heatmap analysis showed XOS has significant impacts on the expression of genes in *S. aureus* ATCC 6538 (Supplementary Figure 3A). Annotation in NR yielded 2,659 genes, including 480 upregulated and 424 downregulated genes with a threshold of | log2FC| ≥ 1 and adjusted *p* < 0.05 (Supplementary Figure 3B). The GO analysis classified all downregulated genes into different terms. Among the top 20 enriched terms, many are related to pathogenesis (e.g., cytolysis, cell killing, and toxin) and quorum sensing (Figure 4A). Partially agreed with GO analysis, KEGG analysis enriched these DEGs mainly (number of unigenes > 20) in pathways of bacterial infectious disease, cellular community, signal transduction, membrane transport, translation, and nucleotide metabolism (Figure 4B). In particular, three of four genes in the *ArgABCD* cluster were significantly downregulated, which are the major components of the auto-inducing peptide (AIP) mediated quorum sensing (QS) process (Figure 4C). The QS is a cross-talk process in which bacteria communicate with each other based on density-dependent signal molecules (36). Inhibiting QS of bacterial population is extremely important in pathogens, as this disables them to initiate most of its virulence activity that helps during infectious disease prevention and control (47, 48). In addition, XOS significantly downregulated many genes involved in *S. aureus* infection, which may lead to decrease of colonization, leukotoxic activity, prevention of membrane attack complex formation, and inhibition of opsonization (Figure 4D). Potential decrease of different toxins during infection should be a result of *AgrABC* downregulation governed by QS, which in fact had been revealed (49). Together with kinds of extracellular enzymes, toxins were considered as important virulence factors playing vital roles in *S. aureus* pathogenesis (50, 51). As demonstrated here (Figures 4C,D), blocking the pathways that regulate toxin production should be of potential in inhibiting or controlling the infection of *S. aureus*, desirably by food ingredients like XOS (52). Of note, a large number of genes encoding ribosomal proteins were also downregulated by XOS (Supplementary Figure 4).

## Conclusion

The report showed the bifidogenic and antibacterial activities of XOS are both dose- and fraction-dependent and restrained within certain species/strains of bifidobacteria. During the production of XOS, control of the composition, especially the proportion for XOS_3_, may be important. During the application of XOS, control of the dose within a certain range is equally important for the bifidogenic activity, at least *in vitro*. To explore the antibacterial activity, it is advisable to add XOS containing higher proportions of fractions with smaller DPs into foods and preferably in combination with probiotics. From aspects of food chemistry and nutrition, this study provided important data for the quality control of XOS production and its effective application. In addition, the study provided novel molecular insights into the application of XOS as an antimicrobial substance or additive.

## Data availability statement

The datasets presented in this study can be found in online repositories. The names of the repository/repositories and accession number(s) can be found in the article/Supplementary material.

## Author contributions

ZS and CL designed the experiment, wrote the manuscript, discussed the results, and finalized the manuscript. ZS and EL carried out the experiments. ZY and XL analyzed the raw data. All authors contributed to the article and approved the submission.

## Footnotes

1
https://cloud.majorbio.com
