# Supplementary material for: Assessment of the bifidogenic and antibacterial activities of xylooligosaccharide
Source: Front Nutr. 2022 Aug 25;9:858949. doi: 10.3389/fnut.2022.858949 (PMC9453197; doi:10.3389/fnut.2022.858949)
Supplement: Supplementary file 1 [file Table_1.DOCX]

#### Supplementary information

# Assessment of the bifidogenic and antibacterial activities of xylooligosaccharide

Zhongke Sun^1, 2, *^, Zonghao Yue ^2^, Erting Liu^3^, Xianfeng Li^3^, Chengwei Li^1, *^

1. College of Biological Engineering, Henan University of Technology, Zhengzhou, 450001, China
2. Institute of Food and Drug Inspection, Zhoukou Normal University, Zhoukou, 466001, China
3. Henan Heagreen Bio-technology Co., Ltd. Zhoukou, 466000, China

*, corresponding authors

Dr. Zhongke Sun, Email: [sunzh@daad-alumni.de](mailto:sunzh@daad-alumni.de);

ORCID: <https://orcid.org/0000-0002-9784-9769>

Prof. Chengwei Li, Email: [lcw@haut.edu.cn](mailto:lcw@haut.edu.cn); Tel: 86-0371-67756888

Post address: No.100, Lianhua Road, Gaoxin District, Zhengzhou City, Henan Province, 450001, China

**TABLE S1 Composition and source of different sugars used in the study**

| Product | ^a^Description | ^b^Composition | Supplier |
| --- | --- | --- | --- |
| D-xylose | D-xylose powder | 99% xylose | Macklin inc. China |
| XOS | Xylooligosaccharide syrup, average DP 2-6 | 95% XOS (42.03% DP 2, 27.9% DP 3, 13.59% DP 4, 14.87% DP≥5) | Heagreen, China |
| XOS_2_ | Xylobiose powder | DP 2, ≥97% | Heagreen, China |
| XOS_3_ | Xylotriose powder | DP 3, ≥97% | Heagreen, China |
| XOS_4_ | Xylotetraose powder | DP 4, ≥97% | Heagreen, China |
| XOS_5_ | Xylopentaose powder | DP 5, ≥97% | Heagreen, China |
| XOS_6_ | Xylohexaose powder | DP 6, ≥97% | Heagreen, China |

a, DP, Degree of polymerization; b, composition was analyzed by chromatographic methods

####



#### FIGURE S1 The fermentability of bifidobacteria on xylose and XOS. (A) growth assayed by reading OD600, (B) *groEL* gene copy number assayed by qPCR, (C) cell count number by counting colonies on MRS agar. Stains of *Bifidobacterium spp.* were inoculated in the basic biochemical broth or the broth supplemented with either 0.25% D-xylose or XOS. Samples were collected after 24 h incubation. All data were mean of three independent experiments assayed in triplicates and normalized to that of samples collected from the basic biochemical broth. Data were analyzed by Tukey’s one-way analysis of variance (ANOVA) when necessary. *B. lactis*, *Bifidobacterium lactis* HN019; *B. breve*, *Bifidobacterium breve* ATCC 15700; *B. bifidum*, *Bifidobacterium* *bifidum* ATCC 29521; ***, *p*< 0.001





#### FIGURE S2 The bifidogenic activity of XOS on three strains. (A) growth assayed by reading OD600, (B) *groEL* gene copy number assayed by qPCR, (C) cell count number by counting colonies on MRS agar. Stains of *Bifidobacterium spp.* were inoculated in the basic biochemical broth supplemented with either 0.25% D-xylose or XOS. Samples were collected after 24 h incubation. All data were mean of three independent experiments assayed in triplicates and normalized to that of samples collected from medium supplemented with xylose. *B. lactis*, *Bifidobacterium lactis* HN019; *B. breve*, *Bifidobacterium breve* ATCC 15700; *B. bifidum*, *Bifidobacterium* *bifidum* ATCC 29521

####
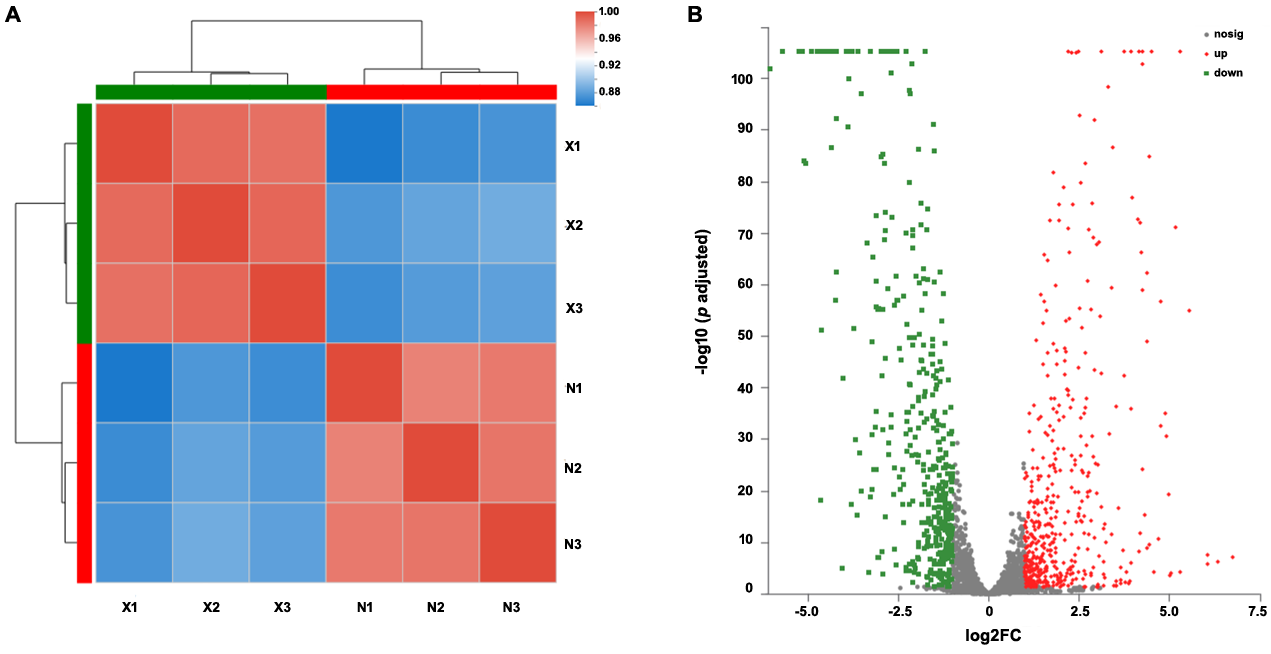


#### FIGURE S3 The gene expression of *Staphylococcus aureus* ATCC 6538. (A) Heatmap dendrogram of all expressed genes in X (X1, X2, X3) and N (N1, N2, N3) groups, (B) Volcano plot of differently expressed genes (DEGs) from the transcriptomes of the control (N) and treatment (X) groups. *S. aureus* ATCC 6538 was grown in LB broth in the absence (N) or presence of 1.0% XOS (X) for 12h. DEGs were screened with the threshold of |log2FC|≥1 and adjusted *p* < 0.05 (X/N). Red dots represent up-regulated genes, green dots represent down-regulated genes, grey dots represent genes without significant difference in the presence of XOS.


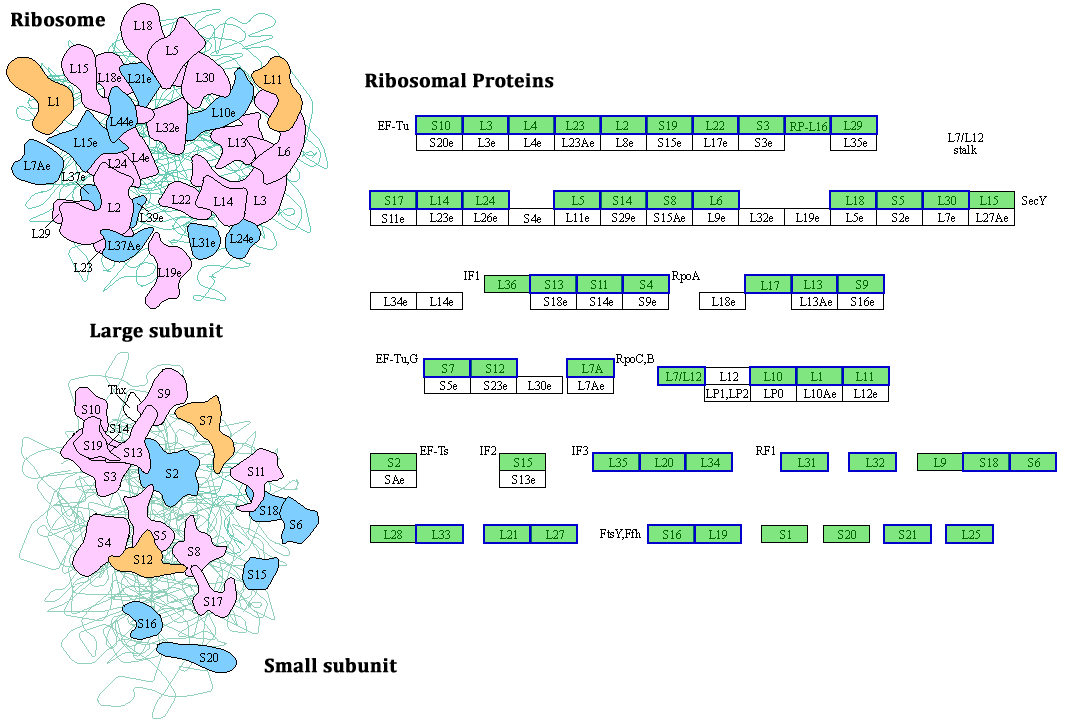


**FIGURE S4 DEGs involved in ribosome in *Staphylococcus aureus* ATCC 6538.** *S. aureus* ATCC 6538 was inoculated in LB broth in the absence/presence of 1.0% XOS. Genes in green box with blue frame are down regulated in the presence of XOS; genes in light green box are expressed but without significant difference. Adapted from KEGG map03010
